# Using embryo models to understand the development and progression of embryonic lineages: a focus on primordial germ cell development

**DOI:** 10.1159/000538275

**Published:** 2024-03-13

**Authors:** Ignacio Rodriguez-Polo, Naomi Moris

**Affiliations:** a The Francis Crick Institute, 1 Midland Road, Somers Town, London, NW1 1AT, UK

**Keywords:** Primordial germ cells, Embryo models, SEMs, Embryonic organoids, PGCs

## Abstract

**Background:**

Recapitulating mammalian cell type differentiation *in vitro* promises to improve our understanding of how these processes happen *in vivo*, while bringing additional prospects for biomedical applications. The establishment of stem cell-derived embryo models and embryonic organoids, which have experienced explosive growth over the last few years, open new avenues for research due to their scale, reproducibility, and accessibility. Embryo models mimic various developmental stages, exhibit different degrees of complexity, and can be established across species. Since embryo models exhibit multiple lineages organised spatially and temporally, they are likely to provide cellular niches that, to some degree, recapitulate the embryonic setting and enable “co-development” between cell types and neighbouring populations. One example where this is already apparent is in the case of primordial germ cell-like cells (PGCLCs).

**Summary:**

While directed differentiation protocols enable the efficient generation of high PGCLC numbers, embryo models provide an attractive alternative as they enable the study of interactions of PGCLCs with neighbouring cells, alongside the regulatory molecular and biophysical mechanisms of PGC competency. Additionally, some embryo models can recapitulate post-specification stages of PGC development (including migration or gametogenesis), mimicking the inductive signals pushing PGCLCs to mature and differentiate, and enabling the study of PGCLC development across stages. Therefore, *in vitro* models may allow us to address questions of cell type differentiation, and PGC development specifically, that have hitherto been out of reach with existing systems.

**Key Message:**

This review evaluates the current advances in stem cell-based embryo models, with a focus on their potential to model cell type-specific differentiation in general, and in particular to address open questions in PGC development and gametogenesis.

## Introduction

The first few weeks of embryo development, when the multiplicity of cell types are generated and tissues and organs become established, are crucial for determining the healthy development of the fetus. The shift from the early epiblast to the post-implantation embryo is accompanied by the transition from relatively homogeneous cells (pluripotency) to an organized embryonic structure with differential cell fates (differentiation). This is the basis for the establishment of both the somatic lineages and the germ line, and the study of these processes has mainly been achieved using animal models, such as the mouse. However, it is becoming apparent that there may be human-specific processes that necessitate alternative approaches, since access to human embryos is often restricted. Additionally, studying development in animal models has limitations including difficulties in tracking development after implantation, and challenges to upscale the experimental setup due to the limited number of embryos per litter [1].

This problem has been partially circumvented by the development of pluripotent stem cell (PSC; see Table of Abbreviations) technologies. Nowadays, we have broad panels of PSC lines (induced from somatic cells or of embryonic origin) generated from species including human and mouse that, in combination with a large body of differentiation protocols, allow us to generate almost every cell type *in vitro* [2], [3]. Most importantly, PSCs usually differentiate following the developmental routes of their embryonic counterparts, allowing us to address questions of fate acquisition in early development. However, most protocols to differentiate PSCs are based on complex cocktails of cytokines and other biophysical clues designed to direct the PSCs to a specific single fate (directed differentiation). These approaches are very effective in generating highly enriched populations of a specific cell type, for example, neurons or cardiomyocytes [4], [5]. Even though directed differentiation protocols have proven useful, they prevent us from studying developmental transitions as they occur in the complexity of the embryo, such as the spatial interactions within particular ‘niches’ or the exquisite temporal feedback control of signalling. Additionally, directed differentiation protocols are usually designed around and hinge on our knowledge of embryo development; where this is lacking, our directed differentiation protocols are likewise limited. Accordingly, to differentiate human pluripotent stem cells (hPSCs) we would like to be able to use human embryonic development as a reference. However, for some cell types we still do not fully understand how fate acquisition happens in the human, and knowledge acquired with animal models is not always translatable [6]. Furthermore, the output cells are often stalled at earlier embryonic states, with limited potential to mature to reach later stages. This has hindered our capacity to understand development and to use such approaches for clinical applications.

One example of a cell type of high clinical interest with directed differentiation protocols that show such limitations, are the primordial germ cells (PGCs). PGCs, the embryonic precursors of the gametes (sperm and egg), are determined during early embryogenesis. Furthermore, the founder PGC population is constituted by only few cells (30-40 cells in mouse [7]), which complicates their characterization at specification stages. After specification, PGCs migrate rapidly and change location through the developing embryo towards the prospective gonads, which makes it especially difficult to track them, in contrast to other embryonic precursor cells whose locations remain relatively static during development. Most of our knowledge of mammalian PGC development has been generated using animal models, such as mice, rabbits and non-human primates, amongst others [8], [9], [10], [11], [12]. However, mouse PGCs (mPGCs) arise with different temporal dynamics, and in an embryonic context structurally distinct from human PGCs (hPGCs) [2]. Additionally, key differences in the mechanics of early PGC development have been identified over the last few years when comparing mouse and primates, including human [6]. For example, even though the differentiation trajectory of primate and mouse PGCs is conserved, some of the key effectors in the transcription factor network differ [4], [6], [13]. In contrast to mouse, SOX17 is key to initiate PGC specification in primates, and PRDM14 is important in both species but with no conserved targets between human and mouse [14]. Another example is the reactivation of SOX2 by mPGC shortly after specification, in contrast to hPGCs [6], [15]. These differences highlight the need to develop species-specific systems when investigating human developmental traits. However, research with human embryos at these stages is restricted both technically and legally [16].

At present, there are several protocols available to generate PGC-like cells (PGCLCs) using PSCs from diverse species, including human [17] mouse [18], [19], rabbit [8], marmoset [20], [21], [22], and macaque [23]. Since the mouse has had a pivotal role in the study of PGC development, mouse *in vitro* differentiation protocols have traditionally been designed based on our extensive knowledge of their *in vivo* trajectory. However, in the case of primates, including human, the lack of equivalent *in vivo* data made it necessary to design protocols based on a combination of knowledge acquired from other species and by trial-and error. This limitation has led to many open questions on the translatability of key aspects between PGCs *in vivo* and *in vitro* counterparts in primate systems, like PGC specification competency, developmental progression potential, or how primate PGCs gain competency to complete gametogenesis. It also hampers our ability to design efficient differentiation methods to acquire cell types of interest, and resulting outputs are often relatively immature or inefficient compared to their embryonic counterparts. A case in point is that no functional human PGCLC differentiated *in vitro* has yet led to mature gametes for fertilisation [24].

Most protocols to generate PGCLCs *in vitro* rely on the generation of large aggregates of PSCs in 3D, termed embryoid bodies (EBs), that are exposed to BMP and other cytokines, to bias towards a PGCLC fate [4], [8], [17], [20], [25], [26]. After specification, clusters of PGCLCs in EBs are isolated to allow further progression, for example, PGCLC expansion, maturation, or in the case of the mouse PGCLCs, fully complete gametogenesis [18], [24], [27], [28], [29], [30], [31]. Even though EBs show sequential phases of gene expression [32], [33], the PGCLCs generated with these protocols arise in a context that does not recapitulate the embryonic spatial environment [34]. Furthermore, available protocols to induce maturation of PGCLCs are based on drastic dissociation and reaggregation with gonadal cells or transplantation to induce an accelerated developmental progression [24], in comparison with *in vivo* PGCs that migrate and establish themselves within a precisely organised spatial environment [24]. All these issues translate into limitations in addressing important questions in hPGC development, including studying cell/tissue interactions, temporal control, patterning, or the interactions of PGCLCs with other cell types. Instead, we argue that researchers should instead consider using engineered systems that share organizational features and dynamics with the real embryo.

For instance, Overeem and colleagues [35] constructed a 2D PGCLC differentiation protocol that, unlike traditional EB differentiation methods, uses low-concentration BMP and extracellular matrix, and results in structures that undergo lumenogenesis and differentiate towards hPGCLCs together with amniotic ectoderm- and mesoderm-like cells [35]. hPGCLCs generated using the traditional EB methods are also accompanied by amniotic ectoderm- and amniotic mesoderm-like cells [13]. However, the addition of extracellular matrix in the Overeem et al. protocol induces drastic changes in the PSCs morphology and in the interactions between them, impacting not only the efficiency of differentiation towards PGCLCs but also the speed at which PGCLCs can mature upon aggregation with mouse fetal gonadal cells [35]. This showcases the power of utilizing developmentally faithful features when designing or engineering new *in vitro* systems. However, such methods can still be described as directed differentiation, since they rely on the exogenous application of signalling factors to bias and promote specific cell type generation. A promising new alternative is to use self-organisation to enable PSCs to direct their own cell type differentiation.

**Fig. 1:**
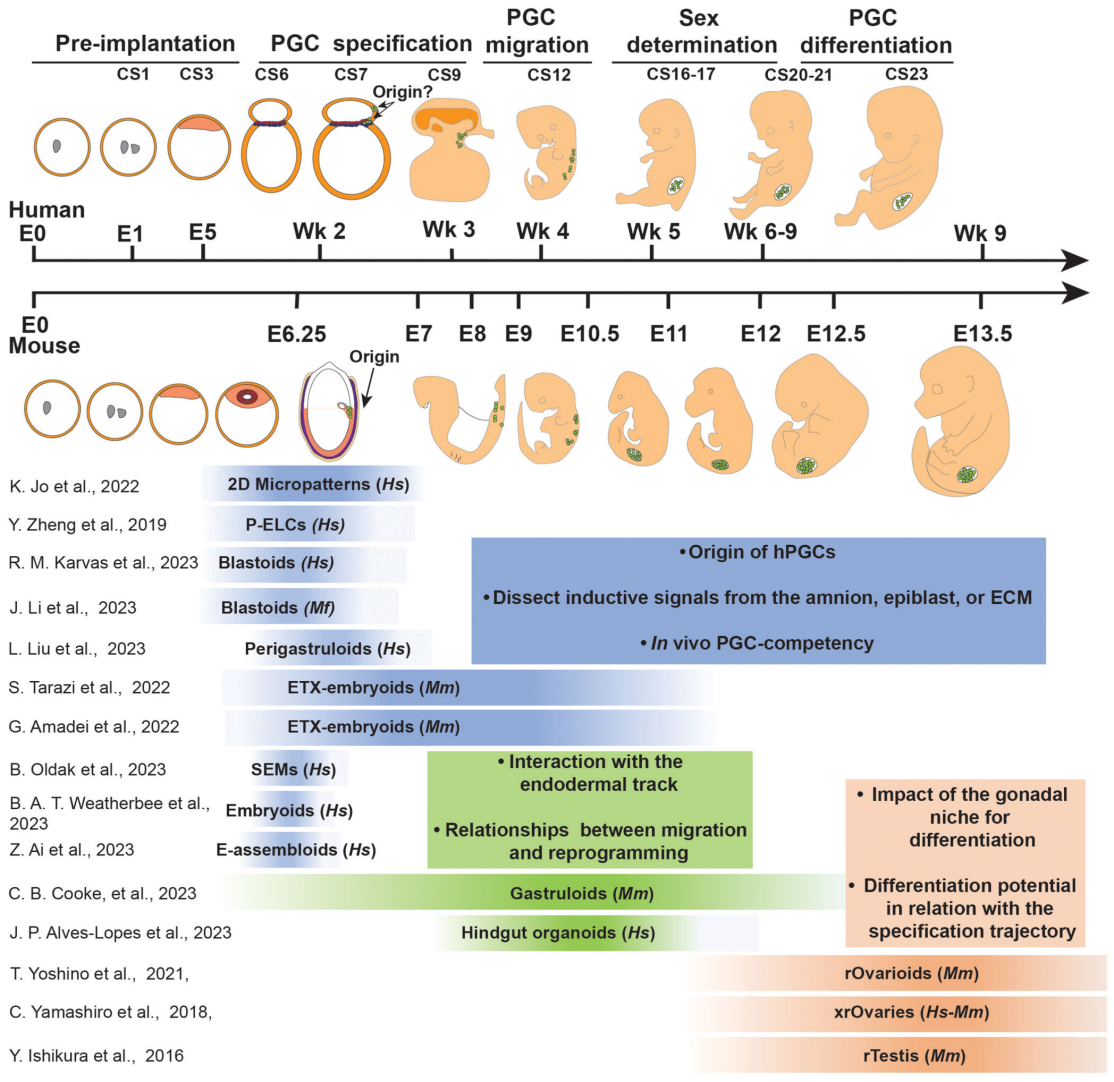
Schematic representation of human and mouse primordial germ cell development (PGC) *in vivo* (upper half of figure) with selected embryo models and embryonic organoids (lower half of figure; human drawings partially adapted from [114]). The different *in vitro* models are positioned with respect to their resembling features along the *in vivo* developmental timeline. Note that this is a tentative placement according to particular features of the *in vitro* systems as they are currently, and may not reflect the potential of each model in the future. Embryo models have been categorized according to their potential to address questions in primordial germ cell specification (blue), migration and epigenetic reprogramming (green), and gametogenesis (orange). Mm, *Mus musculus (Mouse);* Mf, *Macaca fascicularis (Macaque);* Hs, *Homo sapiens (Human*).

The cells of the early embryo possess the inherent capability for self-organization: the ability to generate patterned or ordered outputs from a homogeneous input through localised interactions. In the case of cellular ensembles, this is thought to be achieved through signalling interactions, feedback, cell rearrangements and subsequent cell type specification. *In vitro*, researchers can leverage this embryonic power of self-organisation to enable the generation of increasingly complex structures that recapitulate aspects of embryonic development [34], [36]. These so-called ‘stem cell-derived embryo models (SEMs) or embryonic organoids (depending on whether they present several embryonic tissues or just one, respectively), are derived from PSCs and recapitulate aspects of embryo morphogenesis and lineage specification [34], [37]. These systems are already contributing to our understanding of patterning and cell organization during early embryogenesis [34], [36]. Most of the available models are generated by minimal intervention, using only the strictly essential exogenous inputs necessary to trigger endogenous signalling and the self-organizing properties of PSCs [37], [38]. The minimal intervention rationale may still rely on the addition of small molecules, growth factors and extracellular matrix to generate the different stem cell-based embryo models from PSCs. However, in contrast to directed differentiation protocols, fewer exogenous factors are generally added and in much lower concentrations, so as not to push the cells to differentiate in a specific direction but to trigger patterning and cell rearrangements [36]. Due to the nature of the cells used to develop these systems, most of the models described so far mimic *in vivo* peri-gastrulation events, such as the blastocyst stage or gastrulation towards early organogenesis[34] (shown in Fig 1, and Table 1). However, this is likely to expand further along the developmental window in future. Embryo models have been developed from mouse and human cells providing unprecedented tools to understand embryo development and overcoming several ethical and practical limitations. While many of these embryo models have been observed to contain PGCLCs, it is only now that the value of such systems is becoming clear, such as in exploring the fundamental properties of cell type-specific development, and PGC differentiation in particular [39], [40]. In addition, embryo models present highly varying degrees of complexity, ranges of developmental stages, and reproducibility (shown in Fig 1, Table 1).

Here we review currently available embryo models, analysing their contribution or potential to study PGC development. This comparison should enable examination of the potential of various models in our “toolbox”, in order to exploit the most relevant to answer outstanding questions in the field.

## Embryo models to study PGC competency

Competency has been recently defined as the *“ability to differentiate upon appropriate activation of the inductive signalling pathways”*[41]. Therefore, competency is not a purely cell intrinsic property but it is conditioned by the niche. For example, the capacity of a cell to react to BMP4 depends on the availability of receptors conditioned by cell-to-cell interactions or the gradients of morphogens present in the embryonic tissue [35], [41], [42].

PGC competency has been long under debate. mPGCs arise from the proximal posterior epiblast at the time of gastrulation [11]. In mice, at embryonic day (E) 5.5-6.75 a subset of naïve epiblast cells exit pluripotency [43] and start expressing *Stella* and *Blimp1*, which repress the somatic program and specifies a PGC fate [43], [44]. The cellular subset is localised due to tightly regulated signals, including BMP and WNT, from the extraembryonic ectoderm and visceral endoderm [43], [45]. However, transplantation studies have revealed that the whole epiblast is able to differentiate into mPGCs under particular conditions: therefore, all cells in the epiblast have the potential to become PGCs. PGC specification is therefore restricted to the posterior epiblast *via* signalling-based positional information, such as BMP8b which triggers inhibitory signals in the anterior visceral endoderm [7], [11], [12]. In macaque, PGCs are thought to arise not from the posterior epiblast but from the amnion (˜day 11) shortly before gastrulation starts [10], [46], [47]. In hPGCs, specification takes place between weeks 2-3 after fertilization, however, the exact origin of hPGCs remains elusive. It has been proposed that specification might occur in a dual mode from both the amnion and the posterior epiblast or from a bipotent progenitor population [46], [48]. *In vitro* models of primate PGCLC specification might therefore help to shed light on these species-specific discrepancies and answer this currently open question.

### Using embryo models to examine the starting state of PGCLC-competent cells

*In vitro*, the pluripotent state of the PSCs is believed to be one of the major determinants of PGC competency [4], [12], [48]. Mouse PSCs can be specified to give rise to mPGCLCs by recapitulating the epiblast state and the main signalling inputs[18]. The first step is generating mouse epiblast-like cells (mEpiLCs), that exist in a state between naïve and primed pluripotency [43]. In human, it is possible to induce competent cells to undergo PGCLC differentiation following similar protocols to the mouse, highlighting the high conservation of the differentiation trajectory between mammals [4], [17]. hPSCs can acquire PGC fate from transient pre-mesendoderm (iMeLC/PreME) populations [4], [46], and naïve PSCs (4i conditions) [4]. Additionally, several groups have reported protocols to reset hPSCs into states transitioning between naïve and primed pluripotency that can commit to PGC fate efficiently [13], [49], [50]. Recently Alves-Lopes and colleagues have shown that both capacitating cells (cells transitioning from naïve to primed states) and resetting cells (cells transitioning back from primed to naïve) can differentiate into PGCLCs[50]. Together, this shows the complexity of recapitulating PGC specification *in vitro* using directed differentiation approaches, especially when the starting population is unknown.

**Fig. 2:**
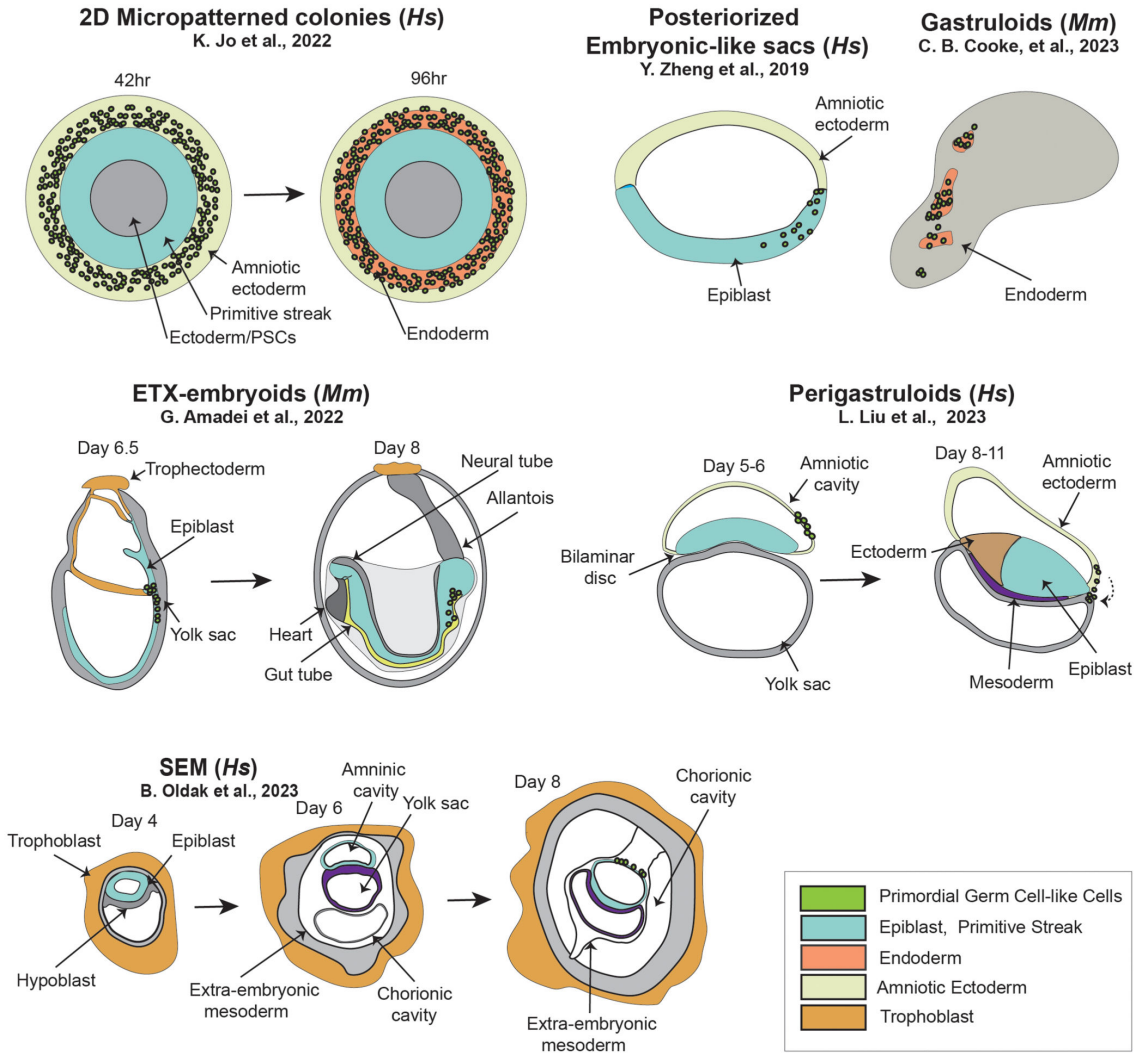
Graphical representation of selected embryo models that show potential to understand competency for primordial germ cell (PGC) specification, and early post-specification stages. The figure includes 2D micropattened colonies, posteriorized embryonic-like sacs, gastruloids, embryonic–trophoblast–extra-embryonic endoderm (ETX-) embryoids, perigastruloids, and SEMs (Stem-cell-based embryo models), schematically representing the different tissues relevant in primordial germ cell development. Mm, *Mus musculus (Mouse);* Hs, *Homo sapiens (Human*).

One of the major advantages of embryo models in this context is that protocols often recapitulate both aspects in parallel: the state of the cells and endogenous signals present in the embryo. Self-organising embryo models are usually generated following minimal intervention, which should facilitate the study of *in vivo* competency. This is not only due to the spatiotemporal analogy to the embryo, but because the signals triggering cell fate acquisition are often endogenous, increasing the probability of an “appropriate activation” [41] including signal intensity and timing, to commit competent cell populations to PGC fate. Various models have proven promising in this regard, including micropatterns, posteriorized embryonic-like sacs, or blastoids, amongst others [39], [40], [51] (shown in Fig 1 and 2). Each has its own advantages and disadvantages when it comes to understanding specification dynamics, features of which will be discussed below.

### Embryo models to explore the signalling mechanism behind PGC fate acquisition

Micropatterned colonies that model germ layer emergence in early development have been pivotal in the move towards self-organising embryo models due to their simplicity and reproducibility [52], [53]. They can be generated from human [52] or mouse [54] PSCs, are cultured on a restricted geometry (shape and size) and exposed to inductive signals that trigger self-organized differentiation in concentric circles. Human PSCs cultured in micropatterns from a primed cell state and exposed to BMP4 for two days present an inner ectodermal/pluripotency domain, followed by a ring of primitive streak markers (BRA+, EOMES+) and an outer ring of amniotic ectoderm- and trophectoderm-like cells [40], [55], [56]. Alongside the extraembryonic ring, this system promotes the appearance of hPGCLCs by 42hr after differentiation onset, in agreement with recent evidence of a concurrent specification of amnion and PGCs in primate embryos from a common precursor cell (+TFAP2A) [48] (shown in Fig 1). Within the micropatterned colonies, the PGCLCs arise in-between extraembryonic cells resembling the amnion, and the primitive streak-like cells, which means they are contextualized in a similar location to hPGCs at this stage [48], [57]. Additionally, the appearance of the hPGCLCs in this location coincides with a response to exogenous BMP restricted to the colony edge due to fewer available receptors and cytokine accessibility in the cells at the core of the colony [42]. This system is highly amenable to imaging (due to its 2D nature and reproducibility) and might provide insights into how signal relay leads to early PGCLC specification. Kyoung Jo and collaborators exploited this system to model hPGC specification and demonstrated that the appearance of PGCLCs, determined by exogenous BMP, is repressed by the reduction of WNT signals [40]. However, by parallel activation of Nodal, PGCLC specification can be rescued. This highlights a primary role of Nodal activation for hPGC specification in a similar way to that which has already been shown in mouse models [58], [59]. This system might potentially be further explored to study early PGC specification at single-cell resolution, including understanding the mechanics behind amnion and PGC emergence, and the regulation of different paracrine signalling gradients to establish PGC fate [40], [42]. However, two major limitations of the model are that the radial symmetry-breaking exhibited by micropatterns has no direct equivalence in the embryo, where the first symmetry-breaking is along a single axis (anteroposterior), and that the flat, 2D and constrained nature of micropatterns might limit their equivalence to embryonic dynamics. For instance, the three-dimensional shape of the embryo, including the juxtaposition between the epiblast and amnion in primates, might be critical to localising morphogen activities and interactions between tissues (rather than mere cell types) necessary for cell specification.

### Using embryo models to explore the origin of the PGCLCs founder population

In humans and non-human primates, the amnion forms shortly after implantation from the epiblast, around day 7 in human and day 9-10 in macaque [57]. One of the arguments defending the potential dual specification of hPGCs from both amnion and epiblast is that during differentiation some amniotic cells seem to conserve post-implantation epiblast signatures. This means they reach an equivalent molecular state (competency) to the cells during the development of posterior epiblast in pre-gastrulation embryos [48], [57]. This makes it expedient to compare PGC competency using primate model systems that have organised epiblast and amnion [57]. One such example is posteriorized embryonic-like sacs (P-ELS, also known as μPASEs [60]), which are generated by culturing 3D hESC aggregates on a microfluidic device [61]. This system exposes the two halves of the aggregate to different controlled signalling environments. Human PSC aggregates cultured in this system spontaneously undergo lumenogenesis, however, if BMP4 is added to one side, the cysts differentiate and self-organize into asymmetrical structures that contain both amniotic ectoderm-like cells (TFAP2A+, CDX2+) and pre-primitive streak epiblast-like cells (BRA+, CDX2+) (shown in Fig. 2). Interestingly PGCLCs are additionally specified during asymmetric embryonic sac development, most likely from the nascent amniotic ectoderm-like cells [60], similar to what is described in macaque embryos [10]. Initially, these PGCLCs are widely distributed in the P-ELS but are mainly located in the incipient amniotic ectoderm-like cell compartment (24hr), and later accumulate in the junction between the amniotic ectoderm and epiblast-like compartments, to finally localise mostly in the posterior epiblast (36hr) (shown in Fig 2, and Table 1). In macaque and marmoset embryos, PGCs show a similar distribution, first observed in the amnion and at a later stage in the posterior epiblast [10], [46], [47]. Since this model is generated by a tightly controlled exogenous BMP gradient, it allows the study of early post-specification PGCs in a context in which the epiblast and the amniotic ectoderm develop and mimic the formation of a bipolar amniotic sac. It is likely that after the initial (exogeneous) polarising BMP4 exposure, the amniotic ectoderm-like cells act as an additional signalling center for the full structure, by localising signalling. P-ELS, and similar models, might therefore help to understand the critical role of the amniotic tissue to establish competency and then commit competent cells to acquire PGC fate.

Amniotic sac-containing embryo models might also be exploited, like micropatterned colonies, to provide clues on the origin of hPGCs by tracking the parallel origin of PGCLCs and the amniotic ectoderm with spatiotemporal resolution due to its increased 3D structural complexity. Furthermore, the technical capacity to engineer morphogen gradients, in the P-ELS model, may also provide possibilities to expose embryo models to a range of localized signalling *stimuli*, to assess their role in PGCLC specification [61]. However, even though micropattern colonies and posteriorized embryonic-like sacs recapitulate some of the key components of the PGC specification niche (for example, the epiblast, early primitive streak and amnion), PGC fate determination occurs in humans around the time of gastrulation (week 2 post-fertilization) [13]. At this time point the embryo starts expressing gastrulation-specific molecular signatures, breaks symmetry, and develops axial organization. This results in early post-specification PGCs being exposed to a changing niche, including embryonic geometry, that is currently impossible to fully model with micropatterns or posteriorized embryonic-like sacs. Alternative embryo-like models may therefore be better able to capture this continuum of stages of PGC development, during and beyond the initial gastrulation stage of development. One example are the gastruloids, which are 3D aggregates of PSCs, that self-organize and subsequently recapitulate key aspects of gastrulation, including gene expression dynamics and multilineage differentiation, and break of symmetry resulting in axial polarization. Gastruloids have been generated from human [62] and mouse [63], [64] PSCs, but a PGCLC transcriptomic signature has so-far only been identified in mouse [62], [65]. Mouse gastruloids are generated from aggregates of naïve cells that, in response to exogenous WNT activation, break symmetry leading to elongated structures with polarized marker expression, around 120hr after aggregation [64], [66], [67]. Interestingly, mouse gastruloids are generated in the absence of exogenous BMP signalling (shown in Table 1) [39]. Recently, Cooke and colleagues demonstrated that PGCLCs in gastruloids recapitulate key aspects of PGC development, including specification and migration [39] (shown in Fig 2). Additionally, by performing perturbation experiments the authors found that neither exogenous nor endogenous BMP was necessary for mPGCLC specification, in concordance with results shown using the EB differentiation method [68]. However, gastruloids do not include any extraembryonic tissues, including amnion, visceral endoderm or extraembryonic ectoderm, meaning that the signalling landscape in a gastruloid is likely to be different to the signalling to determine PGC specification in the embryo [43]. Despite this limitation, gastruloids present the advantage of higher homology to the immediate post-specification PGCLCs niche, including an endodermal tract and maturation-supporting context that enables later-stage PGCLCs to be studied. This system therefore presents a potential model to investigate the co-development of PGCLCs with somatic populations. Future work will also be necessary to develop a human gastruloid equivalent with PGCLC specification potential.

### Potential contributions of embryo models to understand the role of extraembryonic tissues in primordial germ cell specification

Even though amnion, epiblast, and visceral endoderm are likely to be the key signalling regulators of PGCLC specification, the preimplantation embryo has additional extraembryonic complexity that is not captured in the models discussed thus far. In order to fully validate findings on PGCLC specification using more simplified embryo models, it could be helpful to explore more complete models of the embryo. Several such systems have been established that aim to recapitulate the full conceptus of the early embryo. Research in this direction has had to overcome the challenge of differentiating both embryonic and extraembryonic cells from PSCs concurrently. Therefore, most of these models rely on naïve or expanded potential PSCs, and often additionally induce exogenous expression of key transcription factors, to generate the elusive extraembryonic-like states (shown in Fig 1 and Table 1) [69]. Depending on how the extraembryonic tissue is established in the system, it is possible to distinguish between two main techniques: induction and assembly [69]. Models generated following an induction strategy use an aggregate of undifferentiated PSCs and expose them to a signalling cascade that tries to mimic the morula-to-blastula transition *in vivo*, generating structures that resemble the pre-implantation human blastocyst (shown in Fig 1, and Table 1) [70], [71], [72]. The second strategy generates PSC-derived extraembryonic cells separately and later on assembles them with undifferentiated PSCs [69]. Blastoids are models mimicking the blastocyst stage, and were initially developed by assembly [73], but can be generated following both strategies [69] and present all lineages present in the late preimplantation embryo. In contrast, ETX-embryoids (Embryonic–trophoblast–extra-embryonic endoderm embryoids) are generated by assembly of PSCs with trophoblast stem cells and XEN (extraembryonic endodermal) cells. These systems bypass the blastocyst stage and currently mimic later, post-implantation stages (shown in Fig 1, and Table 1) [74], [75], [76], [77], [78].

### Leveraging embryo models for multi-stage study of primordial germ cell development

Embryo models generated by induction are – at the moment - usually restricted to preimplantation stages, which limits their applicability to study PGC specification (shown in Fig 1). However, some groups have been able to push these systems to recapitulate later stages by modulating chemical signalling, inducing their interaction with endometrial cells [70] or embedding them in a 3D extracellular matrix environment, mimicking aspects of the endometrium [70], [79]. Karvas and colleagues [79] used the induction strategy to generate blastoids that were later cultured in extracellular matrix, inducing a progressive expansion of trophoblast lineages and epiblast lumenogenesis, and at later time points, primitive streak formation and early gastrulation[79]. This system also presents a small population of PGCLCs [79], closely recapitulates the environment in which PGCs are specified and also covers a long range of developmental stages (some aspects of CS3-7[79]) (shown in Fig 1). A key advantage of blastoids is that it might be possible to further expand the developmental time course captured by this model. Additionally, further improvements in the non-human blastoid protocols might make it possible to transplant them back to surrogate mothers. Achieving this could provide us with an alternative way of fully recapitulating PGC development in a highly predictive *in vitro* system. Li and colleagues generated macaque blastoids with homologous organization, progression, and degree of complexity to their human or murine counterparts, including the presence of PGCLCs (shown in Table 1) [80]. Interestingly they also transplanted these structures into surrogate macaque mothers, but they failed to further develop. However, this study serves as a declaration of intention for how these models may be used to study dynamic changes during development [80].

Alternatively to blastoids, peri-gastruloids [51] are generated by aggregating expanded potential hPSCs and differentiating them into hypoblast-like cells and epiblast-like cells. These structures form amniotic and yolk sac cavities, specify PGCLCs, and undergo early gastrulation. PGCLCs localise in this model between epiblast-like and amnion-like (TFAP2C+) tissues at day 6, at which point the structures recapitulate aspects equivalent to Carnegie Stage 8 (CS8, days post fertilization 23-25) [51]. Interestingly, hPGCLCs in the peri-gastruloids translocate after specification from the junction between the amniotic cavity to the hypoblast (Yolk sac cavity (PRDM1+/TFAP2C−)) (shown in Fig 2). Peri-gastruloids hold potential to study the PGC dynamics immediately after specification, mimicking the interface between amnion and epiblast, with the addition of a yolk sac-like structure (Shown in Fig 1). Some of the embryo models generated by induction open the possibility to study PGCLC specification in a holistic model with a structure that closely resembles the embryo. However, one of the limitations of the induction strategy is that it relies on exogenous signalling added in the culture medium. These external signals might induce differentiation into extra- and embryonic tissues at different paces generating a developmental timing mismatch [69] or fogging the endogenous signals responsible for PGC specification.

Assembled models partially circumvent these problems allowing the matching of developmental timing of components before aggregating. Additionally, some of the assembloid systems currently exhibit a higher potential to recapitulate post-implantation development towards organogenesis (Table 1). Examples include ETX embryoids [74], [75], [81], [82], [83], and other embryonic assembloids [76], [77], [78], which all present varying degrees of spatiotemporally organized tri-lineages germ layer organisation alongside extraembryonic tissue differentiation. Mouse ETX-embryoids developed by Tarazi et al., [75] and Amadei et al., [74] form an egg-cylinder structure that, upon transfer to an *ex-utero* embryo culture system, leads to incipient organogenesis including beating heart, somite formation and the development of the neural tube [74], [75]. Interestingly, in both assembloid mouse models, mPGCLCs arise at a developmental stage similar to that of the natural embryo (˜E6.5 in the embryo) [74], [75]. In the model developed by Tarazi et al., mPGCLCs are first detected at day 5 of the protocol (Blimp1+Stella−), before they translocate towards the posterior-ventral part of the embryoid (day 6, Blimp1+Stella+) [75]. Similarly, in the Amadei and collaborators model, mPGCLCs arise in the tail region on day 7-8 [74]. Similar assembly-based human embryo model systems have been developed and also exhibit hPGCLCs, but these are much less developmentally advanced than their mouse counterparts, and do not reach organogenesis stages. For example, Oldak and colleagues show that from protocol day 6 onwards their system forms an amniotic sac-like compartment (TFAP2A+ and ISL1+), and around day 8 the epiblast-like structure acquires a disc shape (shown in Fig 2). Concurrent with these morphological changes, a hPGCLC population can be identified [76]. Likewise, Weatherbee and colleagues [77] identified hPGCLCs on day 4 of their protocol, and by transcriptomic profiling identified that their early PGCLC cells express TFAP2A, consistent with the origin of embryonic PGCs from a TFAP2A+ precursor population [40], [48]. E-assembloids [78] are an alternative model generated by Ai and colleagues, generated by assembling naïve hESCs and extraembryonic cells. E-assembloids present hPGCLCs in an organized manner within amniotic tissue or near the junction of EPI-like cells and extraembryonic endoderm-like cells. PGCLCs in these human systems arise concomitantly with amnion-like cell formation in the inner epiblast-like compartments, supporting the idea of a bipotent progenitor cell [8], [48]. In order to fully exploit these models, it will be necessary to further characterise the PGCLC populations, perform deeper molecular profiling and tracking these cells through the complete development of the system. Additionally, the efficiency in generating these models is generally very low, therefore, it will be necessary to increase it to be able to address specific questions, such as those that require screening or large numbers of replicates for statistical power.

### Embryo models to recapitulate cell-ECM interactions mediating PGC specification

Embryo development is not only regulated by cell-to-cell interactions but also by interactions between cells and the extracellular matrix (ECM). ECM production is initiated early in the embryo, and contributes to delineating the boundaries of embryonic tissues and regulating cell and morphogenic dynamics. For instance, in mice, the extracellular matrix secreted by the primitive endoderm plays a crucial role in apicobasal polarization of the epiblast and facilitates lumenogenesis [36], [84], [85].

Similarly, *in vitro*, the addition of ECM to mouse embryonic stem cells and trophoblast stem cells induces the formation of embryo-like structures encompassing an epiblast sac and a trophoblast-like domain [86]. Alternatively, homologous structures can be generated by substituting the ECM with extraembryonic endodermal cells, capable of producing their own matrix. These resultant structures can recapitulate early post-implantation developmental events and, notably, exhibit PGCLCs that are spatiotemporally organized [36], [81].

Likewise, the differentiation of pluripotent stem cells into PGCLCs can be directly influenced by the addition of ECM. This has been demonstrated in 2D human PSC differentiation protocols, where ECM enhances exogenous BMP signalling, enabling the generation of human PGCLCs in 2D with a lower concentration of BMP4 and inducing the formation of luminal structures [35]. Additionally, in a model where human PSC cultures form irregular cysts containing amniotic ectoderm, mesoderm, and PGCLCs, these fates are coordinated through precise modulation of timing and concentrations of 3D extracellular matrix overlay and an ECM gel bed [87]. Notably, the authors establish that amniotic ectoderm cells provide paracrine inductive signals, prompting undifferentiated human PSCs to differentiate into PGCLCs. This emphasizes the critical role of the ECM as a pivotal parameter in the development of embryo models, and the potential of such systems in paving the way for a deeper understanding of the mechanics of human PGC differentiation [87].

From the broad range of human embryo models presented here it is remarkable that in many of them, PGCLCs arise at the interface between epiblast and amnion-like tissue, mirroring their position in the primate embryo [10] (shown in Fig 1, and 2). In addition, several of the models have been shown to recapitulate, to varying extents, the signals required to induce PGC development *in vivo*. It should be highlighted that every model presented here can be exploited to better understand PGC competency and early specification stages. However, it will remain important to carefully select the model according to the specific research question: more reductionistic models might have reduced translatability to the *in vivo* scenario, but be easier to understand, while more complex models may entail laborious or low-efficiency protocols and (particularly in the human case) may additionally present ethical considerations [16]. Therefore, models that present intermediate degrees of complexity, might be ideal for an initial approximation to the research question, that can subsequently be complemented by more complex models as our understanding of PGC competency and specification increases.

## Reconstruction of the post-specification primordial germ cell niche and developmental progression

After specification, mammalian PGCs start migration through the hindgut and dorsal mesentery, between weeks 4-6 in humans [10], [88]. This process is accompanied by drastic epigenetic reprogramming, including chromatin reorganization and DNA demethylation, that allows the competence for gametogenesis upon arrival to the prospective gonads (in human from early week 5) [89], [90]. It is believed that PGCs migrate by combining both passive translocation and active motility [91]. One hypothesis is that PGCs migrate through the hindgut epithelium along the developing nerve fibers [92], [93]. However, the association between these cell types has not been confirmed further, and recent studies in non-human primates conflict with this hypothesis [94]. This process has been especially challenging to investigate due to the difficulty of tracking the PGC population through the embryo. Additionally, migrating PGCs undergo epigenetic and transcriptional reprogramming and this process does not occur in synchrony, which leads to high heterogeneity [95]. This translates into additional challenges to track the molecular progression of the PGC population. Finally, these post-specification stages, when migration and reprogramming occur, have been generally out of reach using *ex vivo* cultured embryos or available *in vitro* models. For example, PGCLCs generated in EBs resemble early post-specification PGCs, and the available protocols to mature them usually require a drastic change of the *in vitro* niche to mimic the gonad environment, thereby completely bypassing the migration stage [96].

**Fig. 3:**
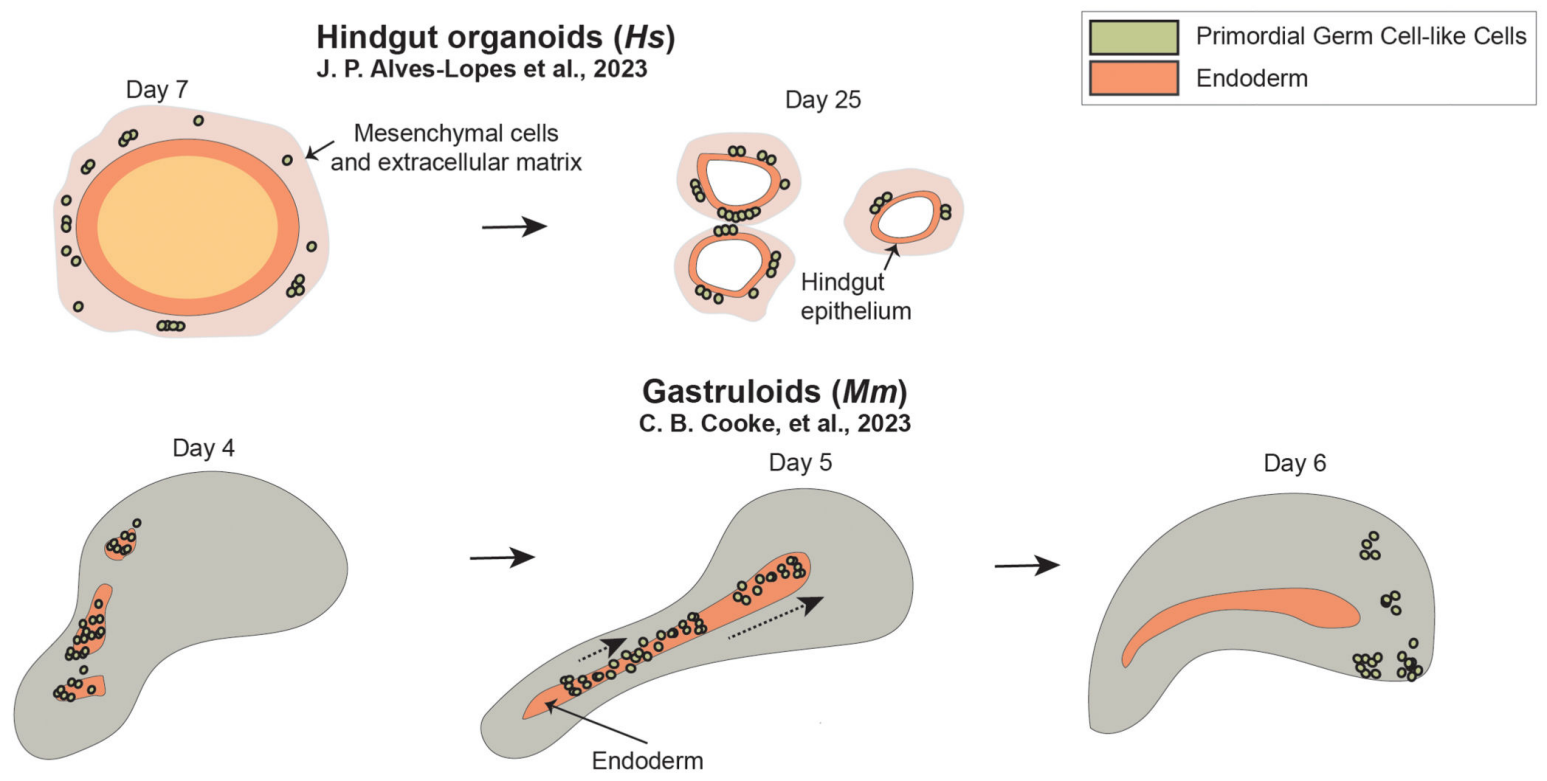
Graphical representation of selected embryo models and embryonic organoids to study primordial germ cell (PGC) post-specification stages, including migration and epigenetic reprogramming. The figure includes human hind gut organoids and mouse gastruloids, including also their initial and final stages before and after maturing *in vitro*. Mm, *Mus musculus (Mouse);* Hs, *Homo sapiens (Human*).

The potential of some embryo models and embryonic organoids, such as micropatterns, gastruloids, hindgut organoids, or blastoids amongst others, to develop *in vitro* and cover a range of developmental stages opens new possibilities to study this embryonic period of PGC migration (shown in Fig 1 and 3, Table 1). Even though most of the models recapitulate features of peri-gastrulation events, some of them already show potential to study migration and epigenetic reprogramming. Embryo models can shed light on processes poorly understood at present, for example, identifying novel drivers of PGC migration to the gonads, how migration and reprogramming processes are interlinked, or how the interaction of PGC with the somatic cells in their migration route impacts the successful directionality during migration and acquisition of epigenetic landmarks.

Some embryo models have already been used to explore PGC reprogramming or the relation of migrating PGC(LCs) with the hindgut mesentery (shown in Fig 1, and 3). In micropatterns, Jo and colleagues showed that hPGCLCs start showing some signs of maturation, including upregulating pluripotency markers in comparison with the undifferentiated cells. Additionally, they start expressing maturation markers such as DPPA3 (Stella) and DDX4 (Vasa), but not DAZL. This is consistent with *in vivo* observations, where DAZL expression has been reported only upon arrival to the gonads [4]. Interestingly, incipient signs of maturation start at the same time as the endoderm (SOX17+ FOXA2+) forms in the colonies between 42-72hr of differentiation, colocalized with the PGCLC population [40]. Even though this system has some potential to study the relationship between PGCs and the surrounding cells, the static nature of this model and the inability to culture this system further make it difficult to exploit for studies into later stages of PGC development. By contrast, mouse gastruloids (120h) present higher structural plasticity and also present an endodermal population of cells organized in a contiguous definitive endodermal tract (BLIMP1+, FOXA2+, SOX17+, CDH1+, and EpCAM+) that distributes through the anterior-posterior axis. Interestingly, mPGCLCs in the gastruloids localize along this structure (shown in Fig. 3) [39]. During their development, mouse gastruloids progressively increase their PGCLC number, initially sporadically arranged through the structure (96h) but increasingly located through the endodermal axis (96-120h), and by 144h most of them have translocated to the anterior and are arranged in clusters (shown in Fig. 3). Cooke et al., hypothesized that PGCLCs might be migrating through the gastruloid, and gastruloid PGCLCs also mimic some features of migrating mPGCs, including the appearance of filopodia-like protrusions [97]. Furthermore, gastruloid-derived PGCLCs mature over time, showing evidence of ongoing epigenetic reprogramming by the overexpression of histone modification H3K27me3 or 5-hydroxymethylcytosine (5hmC) in the PGCLCs at 144hr. Additionally, time course transcriptomic analysis revealed the initial expression of migratory signatures that are progressively substituted in the PGCLC population by mitotic arrest signatures. Finally, PGCLCs express post-migratory markers *Dazl* or *Gcna1* (120-144h) and further comparison to available *in vivo* mPGC data demonstrated that 144h PGCLCs closely resemble *in vivo* PGCs at the mitotic and meiotic arrest stages (E13.5- to E15.5). This system presents great potential to understand the drivers of PGCLC migration and both gastruloids and micropatterns can contribute to the understanding of how the endoderm-PGC(LC) relationship is established, and how this affects their maturation.

The challenges associated with generating an embryo model that recapitulates a range of developmental stages from PGC specification to the end of migration make the use of embryonic organoids an attractive alternative. Embryonic organoids, in contrast to embryo models, recapitulate only one embryonic tissue/organ. This provides the possibility to adjust the developmental stage of the organoids to recapitulate a specific developmental stage without conditioning the development of other embryonic tissues in the system, as would happen in the case of embryo models. One approach to study PGC post-specification stages following this rationale is to generate PGCLCs using the efficient EB-based protocols, dissociate the structures, and then transfer them to an engineered niche that mimics the hindgut. This approach presents a clear separation of the two environments, specification *versus* maturation, but would allow insight into interactions between these different cell types. Recently, Alves-Lopes and colleagues have developed a co-culture system of hPGCLCs with hindgut organoids, established by inducing hPSC differentiation towards endoderm [46] and then into the posterior endoderm hindgut, generating a xeno-free environment for the hPGCLCs [98], [99]. After aggregation of hPGCLCs with the hindgut endoderm-like cells, the authors added extracellular matrix and cultured the system for 23 days. From day 7 onwards, hPGCLCs start to migrate together with mesenchymal-like cells, produced by the organoids, and become randomly distributed through the coculture. After 23 days, hPGCLCs are mainly located on the surface of the hindgut epithelium (CDX2+ and CDH1+), mimicking their *in vivo* location [50] (shown in Fig. 3). Furthermore, the authors describe cytoplasmic protrusions in the PGCLCs. The artificially generated hindgut-like structure allows the maturation of PGCLCs, inducing the upregulation of maturation transcripts, *DAZL* and *DDX4*. Additionally, PGCLCs show evidence of reprogramming by low 5mC or H3K9me2, recapitulating hPGCs between migration and gonadal colonization stages. Interestingly, PGCLCs (generated from resetting precursors) developed in the hindgut organoids at a similar pace to *in vivo* hPGCs, shown by the progressive acquisition of transcriptomic and epigenetic marks. This is crucial to be able to model *in vitro* the spatiotemporal dynamics of hPGCs at post-specification stages. This system provides the possibility to study movement in hPGCLCs in relation to the endoderm, and the relationship between reprogramming and migration processes. Additionally, the generation of PGCLCs and embryonic organoids separately before subsequently integrating them, facilitates the manipulation of one or the other without comprising the full *in vitro* system, for example, generating loss-of-function hPGCLCs or organoid cells. This may be helpful for describing ligand-receptor interactions crucial for the migration of PGCs. However, the use of organoids presents other limitations, for example, the lack of external guidance clues provided by other embryonic tissues or global coordinating signals. Additionally, different migratory stages seem to require specific molecular programs for example the need for CXCR4 expression in the PGCs and SDF1 in the gonadal cells is essential for a successful colonization of the gonadal niche but not essential in earlier migratory stages [100], [101].

In summary, studying the progression of PGCLCs after specification upon the arrival to the gonadal niche has been challenging thus far. However, several embryo models are already able to recapitulate aspects of these developmental stages, including PGC migration, epigenetic reprogramming, or the relation of PGCLCs with the surrounding cells (shown in Fig 1, Table 1). Being aware of how the field is developing, it seems that the generation of novel models or the adaptation of pre-existing ones will be accomplished following two distinct strategies. The first works towards extending the window of developmental stages for existing models like blastoids; fine-tuning the protocols to induce the embryo models to further develop *in vitro*. Alternatively, researchers are creating specific systems to model post-gastrulation stages such as hindgut organoids, that recapitulate aspects of a specific niche corresponding to one or few developmental stages. Both approaches present suitable alternatives with the potential of working together towards a better understanding of PGCs post-specification.

## Reconstitution of gametogenesis *in vitro*

The final hurdle after migration and reprogramming is when PGCs colonize the gonads (week 7–10 in human), proliferate, undergo sex determination and differentiate into pro-spermatogonia or oogonia, in males and females respectively [3]. This process is conditioned by somatic cells in the gonads, granulosa cells in females or Sertoli cells in males. Female sex determination leads directly to the entry to meiosis, while male germ cells undergo mitotic arrest (week 14 in humans, E14.5 in mouse) [102], [103]. The full reconstitution of gametogenesis *in vitro* has been one of the major goals in the PGC field for years [24]. Achieving this milestone could expand our knowledge of the late stages of PGC development and have important biomedical implications. Considering the importance of the gonadal niche in this process, the first attempt in this direction was the transplantation of mPGCLCs into the gonads of newborn mice lacking their own germ cells [18], [43], [104]. The niche in the seminiferous tubules was able to support the development of (XY) mPGCLCs leading to spermatozoa that, combined by intracytoplasmic sperm injection with oocytes, led to viable offspring [18]. Similar approaches have been followed with other species like rat [105] or non-human primates [23]. Sosa and colleagues generated PGCLCs from cynomolgus macaque and performed both xenogenic (macaque PGCLCs into mouse testis) and allogenic (macaque PGCLCs into macaque testis) approaches [23]. PGCLCs in the mouse testis occupied the space next to the basal membrane and differentiated leading to VASA+ and MAGEA4+ but not the spermatogonia marker ENO2. Additionally, these approaches induce only partial epigenetic reprogramming (loss of 5mC but still presence of 5hmC) in the PGCLCs. Following the same approach but transplanting macaque PGCLCs into a macaque testis, the group analysed PGCLC differentiation after 7 months, and equally found MAGE4, VASA positive cells, but no ENO2 positive cells, demonstrating that the cells do not progress to become spermatogia [23]. This progress highlights some of the major challenges to complete gametogenesis *in vitro*. First, it is crucial to allow the PGCLCs to undergo epigenetic reprogramming and gain competency for gametogenesis. Additionally, it is important to generate systems that recapitulate the complexity of the embryonic gonadal niche, match the developmental timing of post-migratory PGCs, and provide cues for PGCLCs to differentiate. Furthermore, there are clear differences between male and female gametogenesis [102], [103], which makes it necessary to generate specialized systems for each sex.

**Fig. 4:**
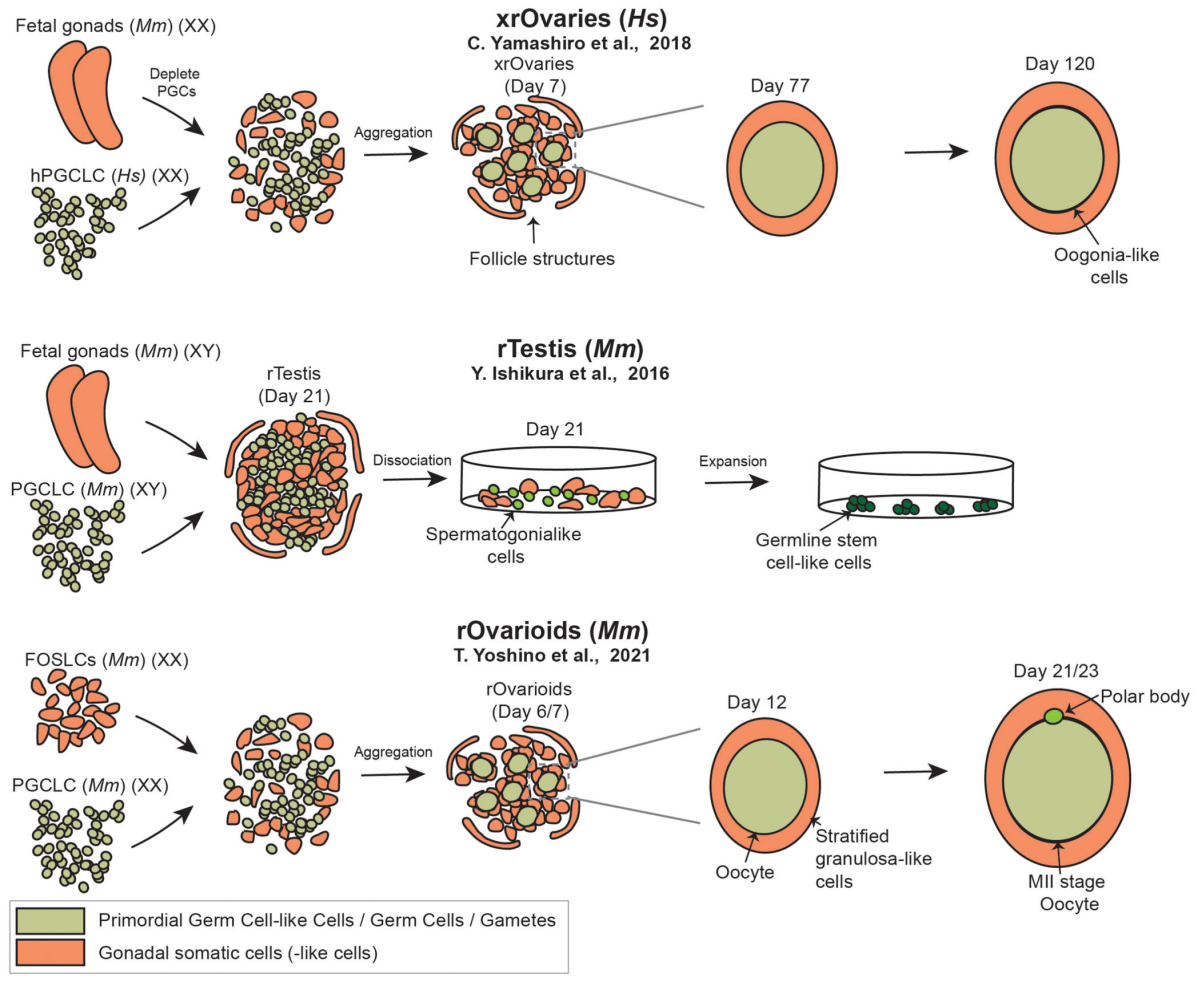
Selected embryo models recapitulating aspects of the entry and progression of primordial germ cell-like cells to gametogenesis. The figure contains a schematic representation of xenogenic reconstituted ovaries, reconstituted testis, and reconstituted ovarioids. Mm, *Mus musculus (Mouse);* Hs, *Homo sapiens (Human*); *rOvaries, reconstituted ovaries; xrOvaries, xenogeneic reconstituted ovaries; rTestes, reconstituted testes*.

We lack embryo models to recapitulate gametogenesis since none of the available systems have yet reached these advanced developmental stages (shown in Fig 1, Table 1). Therefore, to recapitulate this process, we currently rely on the use of PSC-based embryonic organoids. One of the first attempts to model the female gonadal environment *in vitro* was performed by Hayashi and collaborators dissociating mouse embryonic gonadal cells (E12.5) and aggregating them with mPGCLCs to form a reconstituted ovary (rOvary). mPGCLCs in the ovarian-like niche undergo epigenetic reprogramming, including imprinting erasure or X chromosome reactivation, cyst formation and acquire meiosis potential. In order to further push the development of these structures, the authors transplanted the mouse rOvaries into the mouse where they completed maturation and formed later oocytes that, after maturation, could be fertilized *in vitro* to generate fertile offspring [27]. In order to recapitulate the same process fully *in vitro*, Hikabe et al. generated rOvaries and exposed them to different phases of maturation that led first to the formation of mouse primary oocytes and granulosa somatic cells, then germinal vesicle oocytes and finally to oocytes in Meiosis II stage, which can produce viable offspring by *in vitro* fertilization [28]. One of the major limitations of these systems is that they rely on embryonic gonadal cells, which makes them less reproducible and requires the sacrifice of mice. Therefore, subsequent studies developed methods for the *in vitro* generation of gonadal cell-like cells that resemble the E12.5 mouse ovary [31]. These cells can be used for the generation of rOvarioids, that are also able to produce functional offspring (shown in Fig. 4 and Table 1). However, the efficiency of producing oocytes using rOvarioids is lower in comparison with rOvaries [31].

The establishment of homologous human systems has progressed at a slower pace in comparison to their murine counterparts. First human gonadal cells are not often available at fetal stages necessary for such experiments. Additionally, it is not possible to transplant these systems to see how they will continue to develop. Yashimiro and colleagues generated hPGCLCs that were isolated and reaggregated with mouse embryonic ovarian cells to generate xenogenic reconstituted ovaries (xrOvaries) (shown in Fig. 4) [106]. After culture for 70 days, this leds to oogonia-like cells (+DDX4, +DAZL). However, only a small proportion of the initial PGCLC population further progresses. After 120 days, they demonstrated that the hPGCLCs have developed further but have not yet reached meiosis stage. The transcriptomic profile of these cells indicates that they resemble gonocytes (at week 7) and oogonia (at week 9). Additionally, the oogonia show epigenetic reprogramming, including genome-wide DNA demethylation, and reactivation of the X chromosome [106]. In a recent study, Yang and colleagues generated oogonia from hPGCLCs, that were reactivated *in vitro* to continue meiosis in a human isogeneic reconstituted ovary (irOvaries [35], [107]) system, generated by combining hPGCLCs with human fetal ovarian cells from abortus material. After formation and subsequent WNT activation, the authors transplanted the aggregates to mice where they underwent folliculogenesis [107]. Reconstituted ovary systems have allowed the achievement of several milestones in germ cell development, including the full recapitulation of female gametogenesis in mice, and the differentiation of human PGCLCs into oogonia. However, they do not fully recapitulate the structure of the ovary or allow the developmental progression of germ cells without the addition of exogenous factors. Therefore, it will be necessary to continue developing these systems, for example improving the generation of fetal gonadal cell-like cells for human and mouse.

Zhou and colleagues provided the first steps to develop a homologous system to reconstitute full gametogenesis in male mice [108]. They generated mPGCLCs (XY) and cultured them with mouse postnatal testicular cells in 2D. After the cells were cultured for 6 days in meiosis-inducing conditions, PGCLCs differentiated into spermatogonial stem cells and initiated meiosis. The cells were then transferred to specialised medium and haploid spermatid-like cells were generated after 8 days. The spermatid-like cells lead to fertile offspring by intracytoplasmic sperm injection [108]. A similar workflow was followed by Hwang and colleagues using hPGCLCs and embryonic mouse testicular cells, which enabled the differentiation into human proto-spermatogonia-like cells [109]. Alternatively, male mPGCLCs can be aggregated with mouse embryonic testicular somatic cells to generate reconstituted testis (rTestis) (shown in Fig. 4). In this system, mPGCLCs differentiate into spermatogonial-like cells and subsequently into germline stem cells-like cells, that have gametogenic potential [110]. The systems to reconstitute male gametogenesis present similar drawbacks to the rOvaries, and research in this direction will benefit from increased reproducibility, for example generating the somatic cells from the rTestis also from pluripotent stem cells. Additionally, reconstituting some of the structural features of the embryonic testis, like the seminiferous tubules, might help to increase the potential of this *in vitro* system.

Overall, the generation of gonadal organoids made possible the reconstitution of full gametogenesis *in vitro* for mouse, in males and females, and the development of late-stage germ cells in humans (shown in Fig 1). However, there is still potential to further develop these systems. It is necessary to continue refining the methods to generate the supporting cells to generate rOvaries or rTestis, and fine-tuning the culture conditions. Additionally, it could be beneficial to try to reconstitute the embryonic gonads *in vitro* not only at the cell level but also to recapitulate their structural complexity. Finally, it will be desirable to generate more complex multilineage systems, utilise self-organisation principles, or push embryo models to develop until this stage, not only to provide PGCLCs with an *in vivo*-like gonadal niche but also to mimic other tissues key in the regulation of sex determination, like the production of retinoic acid by the adjacent mesonephros [111], [112], [113].

## Conclusions

Stem cell-based embryo models and embryonic organoids are experiencing rapid improvements contributing to our ability to address questions in development that have been out of reach with the current *in vivo* and *in vitro* models. These systems can contribute to our understanding of cell fate acquisition, maturation and differentiation processes due to increasing structural and cell type complexity, endogenous signalling activation and feedback, and their capacity to recapitulate more than one developmental stage. All these features enable the developmental trajectories and temporal dynamics of cells within these systems to echo their *in vivo* counterparts.

Stem cell-based embryo models and embryonic organoids present specific advantages to the understanding of PGC development and gametogenesis. One of these difficulties is that gamete precursors start their developmental trajectory very early in the postimplantation embryo, and continue through a broad range of developmental stages and in various embryonic niches. Some of the available models, like micropatterns, gastruloids, hindgut organoids or reconstituted ovaries have been already exploited in this regard. However, most important is the potential that these systems present to address relevant questions in crucial aspects of PGC development and gametogenesis like the study of PGC competency and origin, epigenetic reprogramming and migration or the differentiation into functional gametes.

Our knowledge of mouse *in vivo* development has cemented the development of first mouse and then primate, embryo models. However, due to key differences in development, it will be necessary to continue refining the developed human systems [16]. Equally important is to develop homologous models for other species, which can be predictive of human PGC development, for example, non-human primates. This will allow us to move back and forth from the *in vitro* to the *in vivo* settings during the development of a novel embryo model, circumvent some ethical issues, and contribute to the reduction of animal experimentation.

Currently, available embryo models mainly recapitulate aspects of early PGC development, including specification and early reprogramming (shown in Fig. 1, Table 1). Even though some of these systems can already model later stages in development, the advancements of these technologies have several limitations. One of them is that the starting point of most of the embryo models are peri-gastrulation stages. This is influenced by the way that these models are constructed, including input cells and minimal exogenous intervention. Additionally, the capacity of these models to progress further is limited by the increasing diversity and complexity of embryonic tissues and size. Furthermore, the short-range paracrine signals regulating early development are progressively substituted by long-range signalling. Additionally, as the embryo progresses, the contribution of maternal factors influencing the development also increases in complexity and diversity. All this makes it challenging to engineer a niche that pushes these models to fully recapitulate later stages in PGC development using only one system. For this endeavour, embryonic organoids present a very attractive alternative, as this system only includes one or few embryonic tissues, which makes them easier to construct. However, such approaches lose the advantages provided by the *quasi-holistic* recapitulation of the embryo provided by stem-based embryo models. One solution could be the generation of intermediate models, between organoid and embryo models that could make recapitulating later stages feasible while retaining developmentally-faithful self-organising environments.

In conclusion, embryo models and embryonic organoids provide reproducible, high-throughput, multi-species systems that can be used to explore cell fate differentiation. These emerging *in vitro* systems will contribute to acquire novel insights into development, including PGC specification and maturation in particular, with promising impacts in the field likely to arise in coming years.

## Supplementary Material

Table of Abbreviations

## Figures and Tables

**Table 1:** List of selected embryo models and embryonic organoids with potential for the study of primordial germ cell differentiation. The table includes the names of the *in vitro* systems, species in which they have been developed, and input cells. Human pluripotent stem cells (hPSCs), Mouse pluripotent stem cells (mPSCs), and macaque pluripotent stem cells (MfPSCs). Additionally, Table 1 includes a selection of embryonic and extraembryonic tissues with special relevance for the study of primordial germ cell development and gametogenesis. Note that the equivalence of an embryo model with a developmental stage is sometimes difficult to assign, and the stages included here are based on the assessment of certain features present in the model.

Embryo models	Species	Input cells	Relevant embryonic tissues	Relevant extraembryonic tissues	In vivo developmental framing	Ref
Micropatterned colonies	Human	Primed hPSCs	Endoderm, Primitive Streak	Amniotic Ectoderm and Trophectoderm	CS3-7	[40]
P-ELS	Human	Primed hPSCs	Pre-primitive Streak/Epiblast	Amniotic Ectoderm	CS3-7	[61]
Gastruloids	Mouse	Naive mPSCs	Endodermal tracts, Epiblast		Gastruloid E5.5-E9.5 and PGCLCs E13.5/E15.5	[39]
Blastoids	Human	Naive hPSCs	Epiblast, Primitive Streak, Endoderm	Diversified trophoblast lineages, Amnion, Extraembryonic Mesoderm	CS3-7	[79]
Blastoids	Macaque	Naive hPSCs	Epiblast, Primitive Streak, Visceral Endoderm	Amnion cavity, Trophoblast	CS3-7	[80]
Peri-gastruloids	Human	Expanded potential hPSCs	Epiblast, Endoderm	Amnion	CS5/6-8	[51]
ETX-embryoids	Mouse	Naive mPSC	Epiblast, Anterior Visceral endoderm, definitive endoderm	Extraembryonic Ectoderm, Amnion	E11	[75]
ETX-embryoids	Mouse	Naive mPSC	Epiblast, Anterior Visceral Endoderm, Definitive Endoderm	Extraembryonic Ectoderm, Amnion	E11	[74]
SEMs	Human	Naive hPSCs	Epiblast, Visceral and Parietal Endoderm	Trophoblast, Amnion	CS6a	[76]
Embryoid	Human	Naive hPSCs	Epiblast, Visceral and Parietal Endoderm	Trophoblast, Amnion	CS6a/b	[77]
E-assembloids	Human	Naive hPSCs	Epiblast, Visceral Endoderm, Primitive streak	Trophoblast, Amnion	CS6a/b	[78]
Hindgut organoids	Human	Naive mPSCs	Posterior Endoderm Hindgut		Weeks 3 to 4	[50]
rOvarioids	Mouse	Naive mPSCs	Ovarian cell-like cells		CS16-23	[31]
xrOvaries	Human	Naive hPSCs, organoids from somatic mouse gonadal cells cells	Ovarian cell-like cells		CS16-21	[106]
rTestis	Mouse	Naive mPSCs, organoids from somatic mouse gonadal cells cells	Testicular cells		CS16-23	[110]
