# Supplementary material for: Using embryo models to understand the development and progression of embryonic lineages: a focus on primordial germ cell development
Source: Cells Tissues Organs. Author manuscript; Available in PMC 2024 Sep 20. (PMC7616515; doi:10.1159/000538275)
Supplement: Table of Abbreviations [file EMS195680-supplement-Table_of_Abbreviations.docx]

**Table of abbreviations and acronyms.**

| **Abbreviations/Acronyms** | **Definitions** |
| --- | --- |
| 2D | Two-dimensional |
| 3D | Three-dimensional |
| BMP | Bone morphogenetic protein family / Also to referred to effectors of the BMP pathway |
| BRA | T-box transcription factor T (T) |
| CS | Carnegie stage |
| d | Days |
| E | Embryonic day |
| EBs | Embryoid bodies |
| ECM | Extracellular matrix |
| EpiLCs | Epiblast-like cells |
| ESC | Embryonic stem cells |
| ETX embryoids | Embryonic–trophoblast–extra-embryonic endoderm embryoids |
| h (prefix) | Human origin |
| h/hr | Hours |
| Hs | *Homo sapiens* |
| iMeLC | Incipient mesoderm like cells |
| irOvaries | Isogeneic reconstituted ovary |
| m (prefix) | Mouse origin |
| micropatterns | Micropatterned colonies |
| mm | *Mus musculus* |
| Mf | *Macaca fascicularis* |
| P-ELS | Posteriorized embryonic-like sacs |
| PGCLCs | Primordial germ cell-like cells |
| PGCs | Primordial germ cells |
| PreME | Pre-mesendoderm |
| PSCs | Pluripotent stem cells |
| rOvary/rOvarioids | Reconstituted ovary/Reconstituted Ovarioids |
| rTestis | Reconstituted Testis |
| SEM | Stem-cell-based embryo models |
| WNT | Wnt/β-catenin pathway |
| xrOvaries | Xenogenic reconstituted ovaries |
| μPASEs | Microfluidic amniotic sac embryoid |
